# Involvement of high-valent manganese-oxo intermediates in oxidation reactions: realisation in nature, nano and molecular systems

**DOI:** 10.1186/s40580-018-0150-5

**Published:** 2018-07-04

**Authors:** Mani Balamurugan, Natarajan Saravanan, Heonjin Ha, Yoon Ho Lee, Ki Tae Nam

**Affiliations:** Department of Materials Science and Engineering, Seoul National University, Seoul, 151-744 South Korea

**Keywords:** High-valent, Mn-oxo, OEC, RNR, Biomimetic

## Abstract

Manganese plays multiple role in many biological redox reactions in which it exists in different oxidation states from Mn(II) to Mn(IV). Among them the high-valent manganese-oxo intermediate plays important role in the activity of certain enzymes and lessons from the natural system provide inspiration for new developments of artificial systems for a sustainable energy supply and various organic conversions. This review describes recent advances and key lessons learned from the nature on high-valent Mn-oxo intermediates. Also we focus on the elemental science developed from the natural system, how the novel strategies are realised in nano particles and molecular sites at heterogeneous and homogeneous reaction conditions respectively. Finally, perspectives on the utilisation of the high-valent manganese-oxo species towards other organic reactions are proposed.

## Introduction

Manganese plays an essential role in many biological processes and undergoes changes in redox state during catalysis and exists in different oxidation states from Mn(II) to Mn(IV). Among many different manganese proteins/enzymes, we are interested in the oxygen evolving complex and ribonucleotide reductases as it involved in generating high-valent intermediates for the catalytic activity. During the past year, significant advances has been made in understanding the biological chemistry of oxygen evolving complex and ribonucleotide reductase (RNR) class Ib and Ic and the unique redox properties of high-valent manganese species involved in the catalytic cycle of these enzymes are essential in the reactivity of these enzymes (Table 1) [1–6]. The oxygen evolving complex containing Mn_4_CaO_5_ cluster, catalyses one of the most important biological reactions occurring in the plants such as light driven oxidation of water to oxygen and protons. Significant progress has been made in understanding the structure and function of the OEC, however, the mechanism of O–O bond formation still remains elusive to experimentalists [7–14]. In many organisms, the manganese containing class Ib and Ic Ribonucleotide reductases (RNRs) are involved in catalysing the conversion of ribonucleotides to deoxyribonucleotides, which is the precursor for DNA replication and repair. RNR class I can be divided into subclasses Ia-Ic is based on differences in structure and metal cofactor. Class Ia RNR is expressed in all mammals and contains FeFe cofactor, whereas class Ib and Ic RNR has only been found in pathogenic bacteria and a dimanganese(III) cofactor in Ib and Mn–Fe cofactor in Ic were identified (Table 1) [15–28]. In addition, mononuclear Mn(III)-oxygen intermediates and Mn(II) ions as Lewis acid catalysts are used by various manganese enzymes of metabolic importance [4–6]. Many issues remain to be answered about the redox properties of enzyme-bound manganese center and the nature of the high-valent intermediate species involved in the catalytic mechanism. So these enzymes have served as inspiration for making model complexes capable of mimicking biological functions or performing synthetically useful organic and other transformations applicable to industrial level. Recent developments in bioinspired manganese chemistry has led to highly active models of the oxygen-evolving complex (OEC), oxygen activating complexes and catalysts for efficient and selective epoxidation, C–H bond oxidation and other organic conversions. Notably, central role of high-valent Mn-oxygen species in reactivity of Mn-dependent enzymes and model systems were investigated towards these reactions. In this review, the key lessons about high-valent manganese-oxo intermediates involved in catalytic cycles of manganese based enzymes are discussed. Based on the fundamental science developed from the natural system, how the novel strategies are realised in molecular sites and nano particles at homogeneous and heterogeneous condition for many different oxidations reactions are presented with previously demonstrated examples.

**Table 1:** High-valent Mn-oxo intermediates involved in OEC and RNR

Name	Biological reaction	Resting state	High valent Intermediates
Oxygen evolving complex	H_2_O splitting	[Mn_4_CaO_5_(Glu)_3_(Asp)_2_(Ala)(His)]	Mn^IV^-O˙/Mn^V^=O
Ribonucleotide reductase (RNR Ib)	Tyrosine radical generation	[Mn_2_^II^(Glu)_3_(Asp)(His)_2_(OH_2_)_2_] with NrdI cofactor	Diamond core Mn^IV^-(μ-O) (μ-OH)-Mn^III^
Ribonucleotide reductase (RNR Ic)	Cysteine radical generation	[Mn^II^Fe^II^(Glu)_4_(His)_2_]	Diamond core Mn^IV^-(μ-O_2_)-Fe^IV^
Ribonucleotide reductase (may be RNR Id)	Tyrosine radical generation	[Mn^II^Mn^II^(Asp)(Glu)_3_(His)_2_] without NrdI cofactor	Diamond core Mn^IV^-(μ-O_2_)-Mn^III^

### Structure and function of oxygen evolving complex (OEC)

**Fig. 1:**
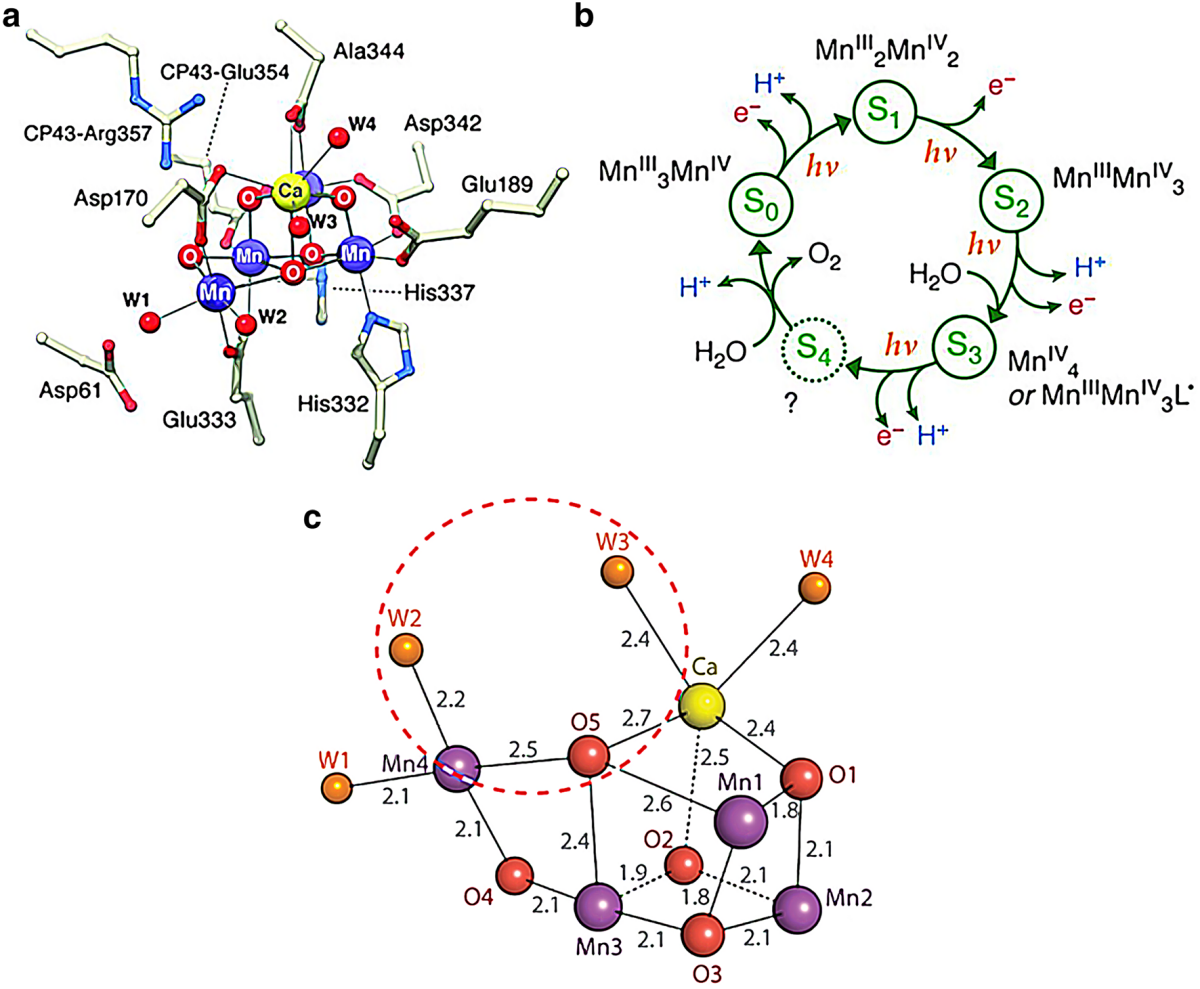
**a** Active site structure of the oxygen evolving complex, a Mn_4_O_5_Ca cofactor with bond lengths [35]. **b** The five intermediate “S” states of the reaction cycle, showing the sequence of electron- and proton-removal steps and the likely oxidation states of the Mn ions in each metastable state. Adapted with permission from [35] (Copyright 2014, AAAS the science society publications. **c** Distances (in Å) between metal atoms and oxo bridges or water molecules [34]. Adapted with permission from [34]. Copyright 2011, Nature publishing group)

Water splitting is one of the most important biochemical reactions on earth in which the light energy is converted into biologically useful chemical energy and molecular oxygen catalysed by oxygen evolving complex in PSII. The water oxidation cycle is catalysed by OEC involves a series of five intermediate states that are known as S states (Kok cycle). The structural and mechanistic information of the OEC, consisting of a Mn_4_CaO_5_ cluster and the structure of PSII has been studied extensively by X-ray crystallography and significant progress has been made in understanding the inorganic and physical chemistry of five different states S0–S4 [10, 29–38]. Nonetheless the identity of the substrate water molecules and the mechanism of the coupling of O–O bond are still elusive. Because most of the collected X-ray crystal structures of PSII using conventional synchrotron radiation have suffered from radiation-induced Mn reduction [39]. Even though, in the 1.9-Å resolution PSII structure the electron densities were clearly separated for all the metal ions and bridging oxygen atoms allowing the clear cut prediction of all the atoms [34, 36]. The oxygen-evolving complex containing an inorganic Mn_4_CaO_5_ cluster in which the four Mn ions and one Ca ion are connected by µ-oxo bridges (Fig. 1) [7–14, 29–38]. The Mn_4_CaO_5_ cluster contains a distorted cuboidal Mn_3_O_4_Ca unit formed by three Mn ion and one Ca ion and four bridging oxo groups. The fourth Mn ion is located outside the cuboidal unit and linked through two oxo–bridges provided by O4 to one of its corners and O5 to the other corner. The Mn_4_O_5_Ca cluster is also surrounded and stabilised by six carboxylate ligands from two aspartate (D1-Asp170, D1-Asp342), three glutamate (D1-Glu189, D1-Glu333, CP43-Glu354) and one alanine ligands (D1-Ala344) and one nitrogen ligand from histidine (D1-His332) residues (Fig. 1b) [34, 36]. Also two water molecules are coordinated to both the terminal Mn ion and Ca ion at the active sites. The Mn_4_CaO_5_ cluster structure is in highly distorted configuration, which enables the cluster to easily undergo structural rearrangements during the catalytic cycle and considered as a salient feature of the cluster. The distorted configuration of the Mn_4_CaO_5_ cluster is originated mainly from the incorporation of calcium ion in cuboidal unit because it alters the Ca–O and Mn–O distances. Among the five oxygen atoms, the bond distances of O1–O4 to their nearby Mn ions in the range of 1.8–2.2 Å and the distance between O5 and its nearby Mn ions is in the range of 2.4–2.6 Å revealing that the O5 is coordinated very weekly with all the nearby Mn ions mainly due to the presence of Ca(II) ion. The incorporation of the Ca(II) ion in the metal cluster for high level distortion in the cuboidal structure and the unique coordination pattern of the μ-oxo-bridged oxygen atoms and their H-bonding interaction with either amino acid residues or water molecules are the important factors contributing to the flexibility and catalytic activity of the OEC [11, 12, 14, 34, 36, 37].

During the reaction initially light energy is absorbed by chlorophyll-*a* (P_680_) and becomes excited and donates one electron to the initial electron acceptor pheophytin moiety, which consequently transfers the electron to the primary and secondary plastoquinone acceptors. The oxidized P_680_^+^ is reduced by a nearby redox active tyrosine residue, which in turn oxidizes a Mn_4_CaO_5_ cluster for water splitting, proceeds through five different states S0–S4 (Kok cycle). After the four sequential oxidation events the OEC advances stepwise through the S0, S1, S2, and S3 states. When the S3 state is advanced to the transient S4 state, O_2_ is spontaneously released and the S0 state is reformed [10, 11, 14, 29–38].

**Fig. 2:**
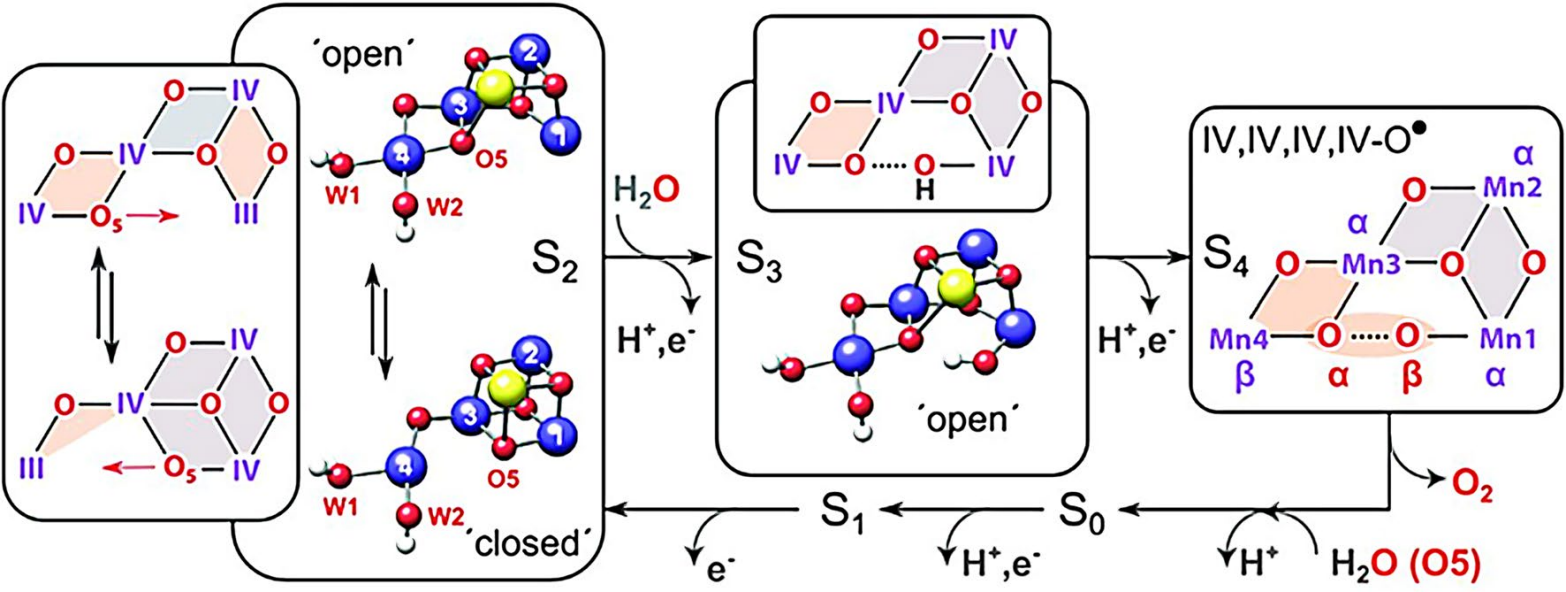
Theoretically calculated structures for the S2 and S3 states and the proposed mechanism of O–O bond formation (Adapted with permission from [35]. Copyright 2014, AAAS the science society publications)

Spectroscopic study and DFT calculations strongly suggesting that the S0 state is the most reduced state in OEC, containing three Mn(III) and a Mn(IV) ion in the Mn_4_CaO_5_ cluster and has a ground spin state of ½ [40–42]. Initially, after the electron transfer cycle the nearby tyrosine radical oxidise one of the Mn(III) to Mn(IV) in the Mn_4_CaO_5_ cluster with concomitant proton transfer and the S1 state contains the Mn oxidation state pattern III, IV, IV, III with spin state of 0 and is diamagnetic [43]. During the second oxidation of the OEC it was observed that no proton is released from the cluster and positive charge is accumulated in the OEC during the transition of S1 → S2^+^ [44, 45]. The S2 state is paramagnetic and has been extensively studied using EPR spectroscopy and two different EPR signal at approximately g = 4.1 is observed and dramatic multiline EPR signal at g = 2 is observed based on the conditions used for the EPR measurement [46–51]. The g = 4.1 and g = 2 EPR signals represents two spin isomers of the S2 state with a ground state of S = 5/2 and S = 1/2 respectively. The Mn oxidation state pattern is IV, IV, IV, III for S = 5/2 state in which the dangler fourth Mn ion is five-coordinated with Mn(III) center and is weakly electronically coupled to the other three Mn(IV) ions in the closed cubane motif. On the other hand, the Mn oxidation state pattern for S = 1/2 state is III, IV, IV, IV in which the Mn1 is a five-coordinated Mn(III) center and all Mn ions are connected by di-µ-oxo bridges resulting in short Mn–Mn distances and promotes antiferromagnetic coupling of the Mn center leading to a low spin state [45, 47, 48, 51]. During the third oxidation of the OEC by the nearby tyrosine radical, the Mn oxidation is coupled with the proton transfer and followed by water coordination and the resulting S3 state’s contain four Mn(IV) centers with six-coordination [35, 52, 53]. Based on the coordination of second water molecule on the cluster many different mechanisms have been suggested and formation of S3 from S2 is still debated. The first mechanism considered the closed cubane S = 5/2 spin isomer and during the oxidation of the Mn4 from Mn(III) to Mn(IV), a water molecule (W3) is transferred from Ca to Mn4 and the water from the hydrogen-bonded network surrounding the OEC occupy the site previously occupied by W3 to Ca [35, 52, 53]. In another mechanism, the open cubane S = 1/2 spin isomer is considered in which a new water molecule added to Mn1 when it is oxidized from Mn(III) to Mn(IV) followed by deprotonation to form a terminal hydroxo ligand on it (Fig. 2).

Inspired from the ammonia binding studies to the S2 state another mechanism is suggested [52, 54–58]. During the oxidation of Mn4 from Mn(III) to Mn(IV) a water molecules from Mn4 is inserted between Mn4 and Mn3 and the water molecule (W1) coordinated to Mn4 center replace the site previously occupied by W2 and a water molecule hydrogen bonded with O4 around is coordinated to Mn4 in the site occupied by W1 [35, 52, 56, 59]. Experimental data for the S4 and the transition of S3 → S0 states are limited, however, computational studies are supporting the mechanism of O–O bond formation and this transition consist of O_2_ formation and release along with two protons and binding of a water molecule to the Mn_4_O_5_Ca cluster. Two different isoelectronic intermediates species such as Mn^IV^–O· radical or a Mn^V^=O species are suggested to be involved in the S4 state and their involvement is still debated. An oxo–oxyl radical coupling mechanism for O–O bond formation has been supported by extensive computational studies in which the Mn^IV^–O· radical species couple to a µ-oxo bridge [35, 60–64]. Based on the studies from inorganic water oxidation catalysts, the water-nucleophile attack mechanism for O–O bond formation has been suggested in which the highly electrophilic Mn^V^=O species attack the oxygen on the water [8, 65–67]. However, to date, no experimental evidence has been collected to support either the oxo–oxyl radical mechanism or the water-nucleophile attack mechanism in OEC. Even though most of our understanding of O–O bond formation arrived from computational studies the chemistry of OEC is supportive in the design of synthetic catalysts for efficient water oxidation reaction. More study on OEC is crucial to understand the mechanism and how OEC stabilised in the protein pocket and utilise the high-valent Mn-oxo intermediates towards hydrogen abstraction and electron transfer reactions in a classy manner to effect the oxygen evolving reaction.

### Ribonucleotide reductases (RNRs)

**Fig. 3:**
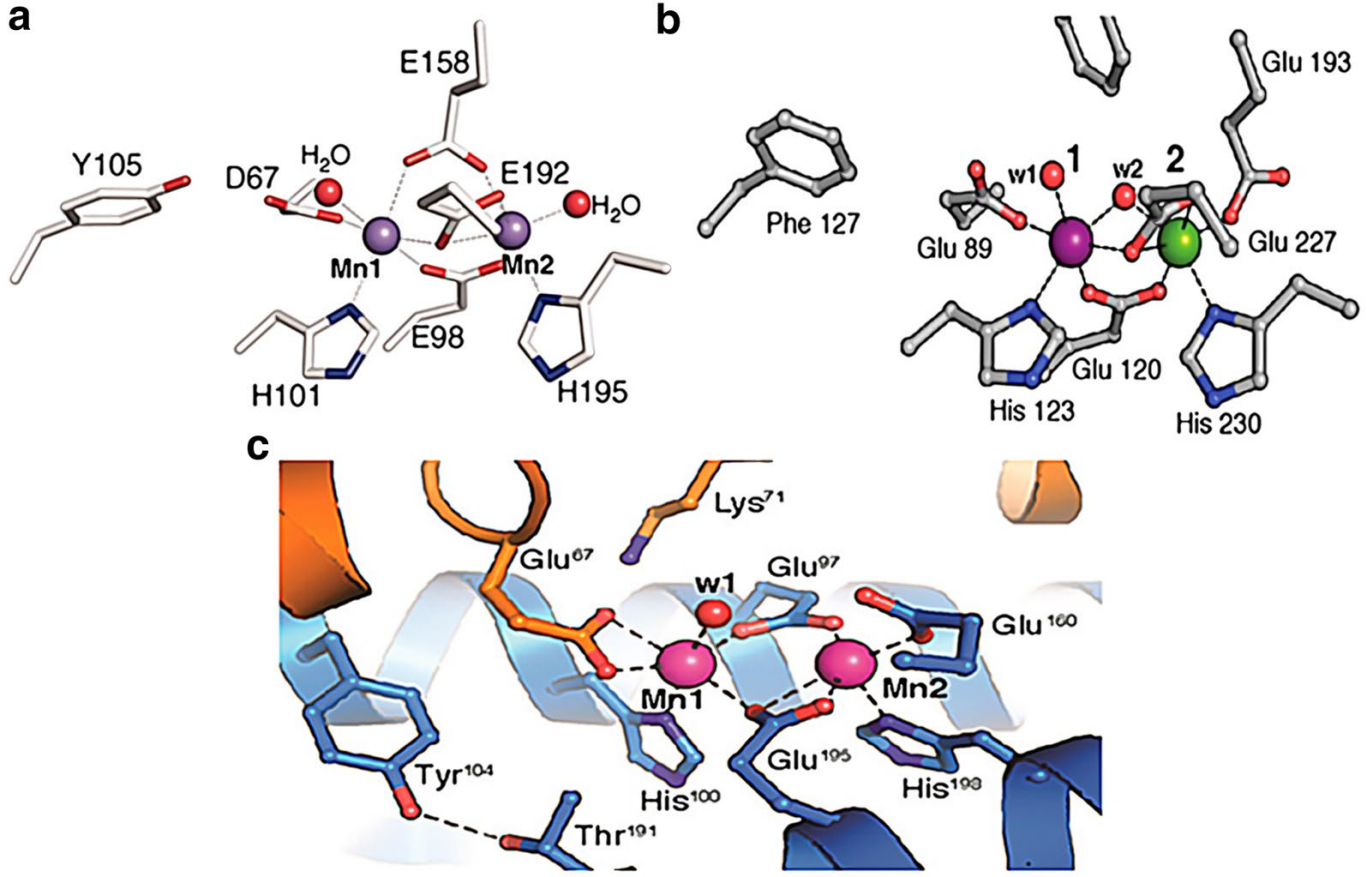
Ribonucleotide reductase class Ib, Ic and may be Id. **a** X-ray crystallographic structure of ${\text{Mn}}_{2}^{\text{II}}$-NrdF cofactor. Adapted with permission from ref [23]. Copyright 2014, AAAS the science society publications. **b** X-ray crystallographic structure Mn^II^Fe^II^ cofactor (Adapted with permission from ref [82]. Copyright 2013, ACS publications. **c** Adapted with permission from ref [87]. Copyright 2018, ACS publications)

In all organisms, Ribonucleotide reductases (RNRs) are the key enzymes involved in catalysing the conversion of ribonucleotides to deoxyribonucleotides, the precursor for DNA replication and repair. In class-Ib RNR from *Corynebacterium ammoniagenes* contain a MnMn cofactor and class-Ic RNR from *Chlamydia trachomatis* (*Ct*), contain a MnFe cofactor in subunit R2, instead of an FeFe cofactor plus a redox-active tyrosine in class-Ia RNRs [15–28, 66, 67]. The 1.65 Å resolution crystal structure of ${\text{Mn}}_{2}^{\text{II}}$-NrdF contains one monomer per asymmetric unit with the presence of two Mn^II^ sites (Fig. 3) with a Mn–Mn distance of 3.7 Å [23]. Mn1 is coordinated by His101, Asp67 and a terminal water molecule and Mn2 is coordinated by His195 and a terminal water molecule. Three glutamate residues (Glu98, Glu158, and Glu192) bridge the two metals in a manner previously not observed in RNRs and related carboxylate-bridged diiron enzymes [23, 68]. The location of the two interacting solvent molecules at Mn2 could easily accommodate by molecular oxygen [22]. These waters may dissociate, allowing the oxidant to initially bind terminally to Mn2 in this position, by analogy to the proposal for H_2_O_2_ binding to the structurally related Mn catalases [69].

Class Ib with dimanganese cluster participate in the generation of tyrosyl radical in R2 unit and the reduction of the catalytic site in R1 [70]. However the di-manganese cluster adopts a somewhat similar coordination environment to that of methane monooxygenase hydroxylase unit (MMOH) [23, 25, 70]. Unlike class Ia and Ib, in class Ic the tyrosyl radical site is replaced by a phenylalanine residue and all the carboxyl ligands to the metal ions are substituted by glutamate residues. By use of Mössbauer, EPR and extended X-ray absorption fine structure (EXAFS), it has been shown that in class Ic R2 proteins, a mixed metal center Mn^IV^/Fe^III^ is the metal cofactor [19–22, 71, 72] acting as source of the oxidation catalyst, capable of generating the thiyl radical in R1.

**Fig. 4:**
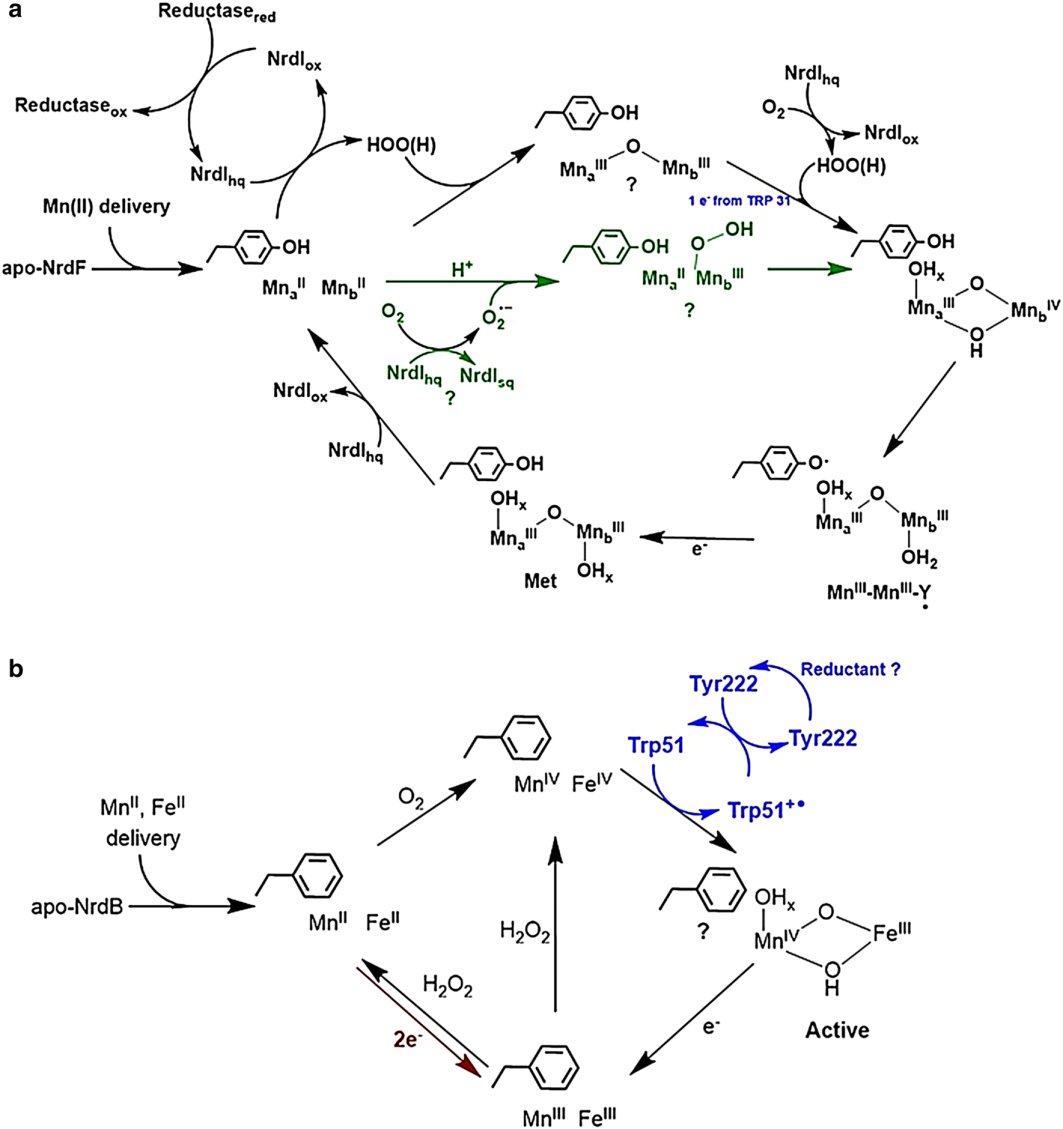
The proposed reaction cycles for the generation of the tyrosyl radical in **a** class Ib and **b** Ic RNR suggested by Stubbe. Redrawn from ref [73]

The mechanism of oxygen activation in the class Ib RNR is derived from EPR spectroscopy along with X-ray crystallography of the protein. RNR utilise oxygen activation mechanism and attain high-valent states and producing tyrosine radical. Two different mechanistic pathways for dimanganese and radical cofactor assembly have been proposed [23–25, 73]. But in both mechanisms the first step involves oxidation of the Mn^II^/Mn^II^ to Mn^III^/Mn^III^ by H_2_O_2_ is considered. In the second step, the oxo bridged Mn^III^/Mn^III^ dimer converted into diamond core Mn^IV^/Mn^IV^ intermediate and it is having sufficient oxidizing power to extract one electron and one proton from the tyrosine residue [23–25, 73]. However different coordination mode of the peroxide ligand is proposed. Cox and co-workers suggested that the HO_2_^−^ or H_2_O_2_ coordinates to both the manganese atoms through only one oxygen atom along with the shift of the bridging carboxylate ligand to produces an oxo-bridged Mn^III^/Mn^III^ complex [25]. After that the second H_2_O_2_ replace a water ligand at Mn_a_ closest to the tyrosyl radical (Fig. 4) and produce a bridging hydroperoxide species of Mn^III^-O-Mn^III^. The protonation of the bridging hydroperoxide and release a water molecule leading to a second two-electron oxidation process to produce the highly unstable Mn^IV^/Mn^IV^ transient species followed by formation of a more stable Mn^III^/Mn^IV^ state by oxidizing a nearby tyrosine residue. Both Mn^IV^/Mn^IV^ and Mn^III^/Mn^IV^ have the oxidizing power to extract an electron from the tyrosine cofactor [25]. But Cotruvo and Stubbe proposed that HO_2_^−^ or H_2_O_2_ replaces a water ligand on Mn_b_, which is slightly away from the tyrosine radical site and forms a bridging hydroperoxide between the two Mn(II) ions with concomitant rearrangements of carboxylate oxygen (E202). After that the oxidation of the Mn^III^/Mn^III^ cluster by the bound hydroperoxide leads to release of a water molecule to produce the diamond core Mn^III^/Mn^IV^ intermediate rather than a Mn^IV^/Mn^IV^ intermediate. Also from the crystal structure of the R2F subunit with NrdI cofactor from class Ib *E. coli*., Cortuvo and Stubbe proposed that the flavodoxin like protein NrdI (NrdIhq) is an essential component for the making the HO_2_^−^ required in the reaction cycle with di-manganese [23, 24, 69, 72]. Class Ic RNRs also utilise the dimetal cluster but instead of dimanganese in class Ib it is utilising MnFe cluster and producing cysteine radical rather than a tyrosine radical [19–21, 74–86]. In Chlamydia trachomatis (Ct) enzyme the Mn^II^/Fe^II^ complex reacts with O_2_ to form a Mn^IV^/Fe^IV^ intermediate followed by one electron reduction to produce Mn^IV^/Fe^III^ cofactor, in which the Mn^IV^ is the oxidant in the active state. The Mn^II^/Fe^II^ cofactor can also react with H_2_O_2_ and converted to the to the active Mn^IV^/Fe^III^ state in two steps through Mn^III^/Fe^III^ and Mn^IV^/Fe^IV^ intermediates [77]. At the position of the tyrosine radical center in Ia/b proteins instead the Ic subunits have phenylalanine; and obviously it is considered as the characteristic of this subclass [19–21, 74–82]. Interestingly, a ribonucleotide reductase (RNR) from *Flavobacterium johnsoniae* (Fj) differs fundamentally from all the class Ia–c RNRs and it is assigned to a new subclass Id. Even though, its active site is similar to class Ib counterparts it does not require the oxidant supplying flavoprotein (NrdI) needed in Ib systems for superoxide (O_2_^−^) activation and it can scavenging the oxidant from solution itself [87]. Interestingly, in all the manganese containing RNR subunits nature utilise the high-valent Mn(IV) species to initiate the hydrogen abstraction from their counter radical-harbouring domain for the further reaction, in making the biologically important conversion such as deoxyribonucleotide from ribonucleotide. Also based on having with or without the flavoprotein counter part, the Mn_2_ cofactor activate the active oxygen species or molecular oxygen to carry out the hydrogen atom transfer reaction (HAT).

### High-valent manganese-oxo intermediates at nano sites

Natural systems successfully utilise the high-valent manganese-oxo species for robust catalytic oxidation, inspired the scientific community. Several attempts have been made to understand and utilise, the novel strategies employed by the natural system. Numerous reports are available for many different oxidation reactions utilising manganese oxide nano particles in the literature, however, studies which involves the characterisation and mechanism of high-valent Mn-oxo intermediates in the catalytic cycle are scarce. Notably, various kinds of manganese oxide nanoparticles are studied for water oxidation reaction and the importance of the distortion in the mixed valent manganese centers in the Mn_4_CaO_5_ cluster for the oxygen evolution reaction is realised on the surface of the manganese oxide nano particle [88–103].

**Fig. 5:**
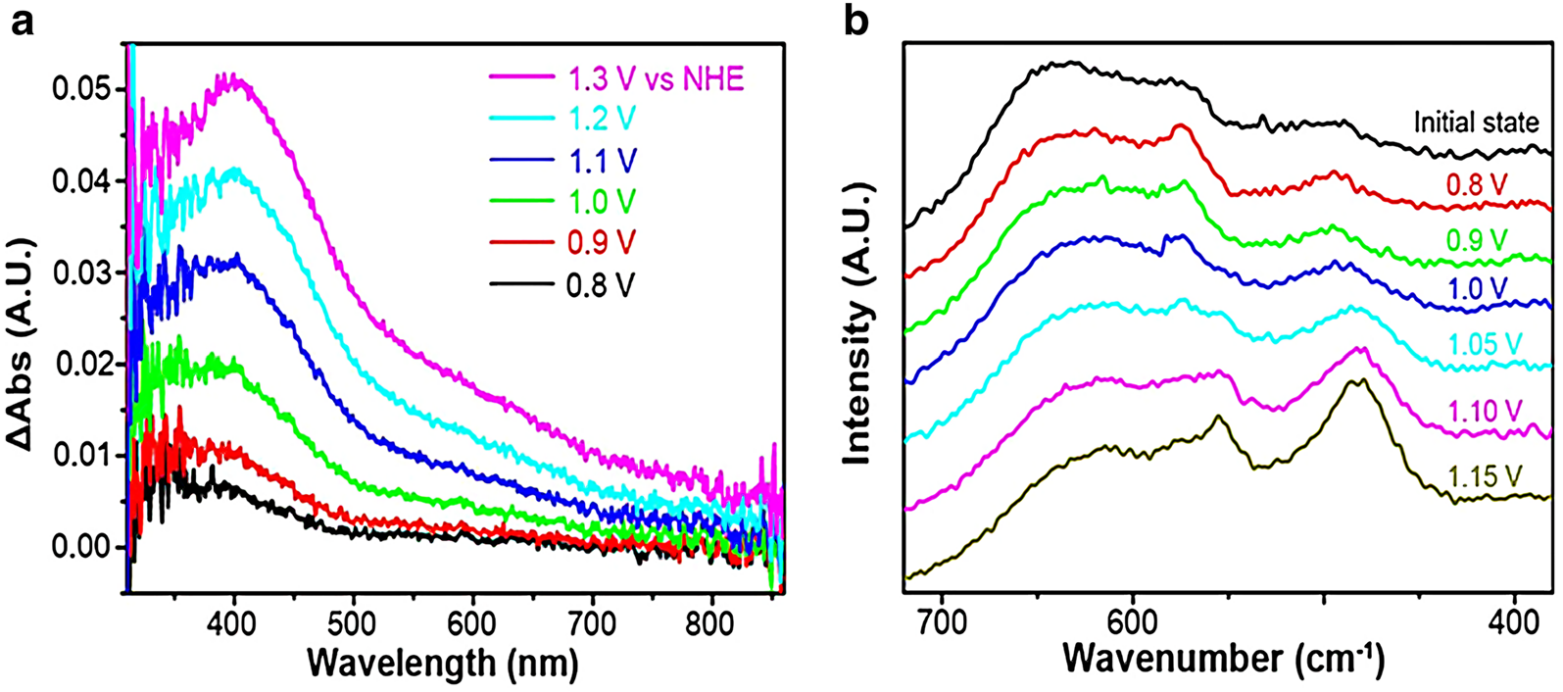
**a** *In*-*situ* UV–Vis difference spectra of the MnO NPs based on the applied potential. **b** *In*-*situ* Raman spectra of the MnO NPs at various potential collected during bulk electrolysis [105] (Adapted with permission from ref [105]. Copyright 2017, ACS publications)

Kurtz et al. studied the biomimetic oxidation of water and shown that the photocatalytic activity of the calcium manganese(III) oxide hydrates (CaMn_2_O_4_·xH_2_O) is superior than the manganese(III) oxide particles (α-Mn_2_O_3_) and related the importance of elemental composition of the Mn_4_O_x_Ca core of the OEC in the activity [86]. The Jaramillo group proposed that the higher OER activity of mixed-valence MnO_x_ film is depends on the high-valent manganese distribution on the oxide material [89, 90]. Also, the effect of hetero atom on the catalytic surface are studied in the MnO_x_/Au-GC composite, synthesized by adding Au to MnO_x_, displayed a surprisingly high enhancement in catalytic performance compared to pure MnO_x_ catalysts [91]. He and Suib group used a similar approach in a photochemical water oxidation of the gold-nanoparticle modified MnO_2_, evidenced from XANES, addition of a small amount of gold to the MnO_2_ surface partially reduces the Mn species to create a mixed valence state thereby enhance the catalytic activity compared to pristine MnO_2_ [92]. Dau group observed the formation of disordered Mn^IV^O_2_ motif for high catalytic activity at neutral pH [93]. Navrotsky group theoretically verified the effect of mixed valence state on OER performance. Also they experimentally demonstrated that among the four different manganese compounds, CaMnO, Mn_2_O_3_, MnO_2_, and Mn_3_O_4_, the mixed-valence CaMnO (Mn^3+^ and Mn^4+^) exhibited the highest catalytic activity [94]. Nocera and co-workers reported the oxygen evolution activity of electrodeposited MnO_x_ films and explains how the original birnessite-like MnO_x_ (δ-MnO_2_) undergoes disordered phase change during OER cycling to exhibit high activity [95]. The Driess group synthesised an amorphous MnO_x_ compound using chemical oxidant ceric ammonium nitrate (CAN) and proposed that the change in oxidation state of the amorphous MnO_x_ compared to the initial crystalline MnO is the key for high reactivity and using EXAFS analysis they insisted that the active site of amorphorized MnO_x_ resembles that of the Mn_4_Ca cluster [96, 97]. They also synthesised amorphous MnO_x_ layered Mn_3_N_2_ particles by molecular approach, in which the layer generated by stepwise oxidation of Mn^2+^ to Mn^4+^ and documented as the real active sites. Importantly, they argue that the Jahn–Teller distorted Mn-O bonds generated by Mn^3+^ assisted Mn^4+^ for binding O–O with appropriate strength is facilitating the OER [98]. Our group reported many different manganese oxide materials for OER [99–106], among them the nanosized Mn oxide catalysts display outstanding catalytic activity under neutral conditions [102, 105]. The sub-10 nm-sized monodispersed MnO nanoparticles showed unexpectedly high OER performance compared to the well-known catalysts Co-Pi and MnO_x_. The stability of Mn^3+^ intermediates on the nanosized oxide surface was also significantly improved during catalysis. Very interestingly, we successfully demonstrated that the 10 nm size MnO stably generate the Mn(III) species via proton-coupled electron transfer pathway. Furthermore, we spectroscopically characterised the reaction intermediate Mn^IV^=O species using in situ UV–Visible and resonance raman analysis during the catalysis (Fig. 5) [105]. Raman spectra of the MnO NPs during electrolysis at constant potentials showing the characteristic Mn(II)–O stretching vibration (A_g_) and Mn(III)–O stretching (E_g_) modes as broad shoulder bands around 640 and 575 cm^−1^ respectively. Upon increasing the applied potential to 1.05 V vs NHE, new Raman peaks appeared at approximately 555 and 480 cm^−1^, with corresponding decrease in intensity of the Mn(III)-related bands. The shift in the Raman values and relative intensities of the generated peaks were assigned to the stretching vibration of Mn(IV)–O species. We also identified the generated reaction intermediates using in situ diffuse transmission UV–vis analysis. Initially, the MnO NPs exhibited two bands in the UV regions at approximately 350 and 380 nm, corresponding to O^2− ^→ Mn^II^ and O^2−^ → Mn^III^ ligand-to-metal charge transfer along with several weak peaks originating from d–d transitions in the visible region. Upon increasing the applied potentials at 0.1 V intervals, two distinct absorption bands in the regions of 400 nm and 600 nm were identified. The origins of the peaks were assigned to Mn(IV) species, by matching with broad peak in the region of 400 nm and shoulder peaks at approximately 575 and 700 nm of Mn(IV) in MnO_2_ [105]. In most of the water oxidising manganese oxide catalysts the generation of the distorted structure on the surface by generating the mixed valency by various thermal, chemical and electrochemical methods are realised with respect to the OEC in which the incorporation of Ca ion and mixed valency plays a crucial role in distorting the structure and function the OEC.

### High-valent Mn^IV^-oxo Intermediates at Molecular Sites

Although manganese is the Nature’s choice for the catalytic oxidation of water in photosystem II, there are only a few reports of synthetic manganese compounds which are able to catalyze this reaction. However, various manganese complexes are reported for the oxygen atom transfer (OAT) and hydrogen atom transfer (HAT) reactions and realised the involvement of high-valent Mn(IV)-oxo species in the catalytic cycle. The first characterized mononuclear manganese(IV)-oxo complexes with porphyrinic ligands were reported by Groves and co-workers. The reaction of manganese(III) complex [(TMP)Mn^III^Cl] with peroxy acid produced a stable [(TMP)Mn^IV^O] and [(TMP)Mn^IV^O(OH)] species, capable of transferring their oxo group to olefins to produce epoxides [107–109]. The manganese salen complexes (Jacobsen catalysts) have been extensively studied as catalysts for the oxidation of olefins to the corresponding epoxides, in which they identified the formation of [(Salen)Mn^IV^(O)] species using EPR and NMR upon interaction of [(Salen)Mn^III^] with *m*-CPBA or PhIO and proposed as the reactive intermediate involved in the catalytic cycle [110, 111]. Later, Yin and co-workers isolated a non-heme monomeric Mn(IV)-complex [(Me_2_EBC)Mn^IV^(OH)_2_]^2+^ (where Me_2_EBC = 4,11-dimethyl-1,4,8,11-tetrabicyclo[6.2.2]-hexadecane) with two hydroxo ligands and employed as catalysts for epoxidation reaction with and without peroxides [112, 113]. A novel manganese(IV) peroxide intermediate, [Mn^IV^(Me_2_EBC)(O)(OOH)]^+^, was captured as the third kind of active intermediate responsible for epoxidation and the *tert*-butyl peroxide adduct of this manganese(IV) complex was also detected by mass spectroscopy under catalytic oxidation conditions [114]. Also they studied the pH dependence HAT reaction rates of the organic substrates (xanthene, fluorene, 1,4-cyclohexadiene, 9,10-dihydroanthracene) using Mn^IV^(OH)_2_^2+^ (BDEOH = 83.0 kcal/mol) and Mn^IV^(O)OH^+^ (BDE_OH_ = 84.3 kcal/mol) species and presented a different hydrogen atom abstraction rates [115]. Interestingly, Nam and co-workers reported the first example of reversible O–O bond cleavage and formation between the in situ generated Mn(IV)-peroxo and Mn(V)-oxo corroles supported by various spectroscopic methods such as UV–vis, EPR, ESI–MS and XAS/EXAFS analysis [116]. Later they studied the reactivity of various Mn(IV) species [Mn^IV^(BQCN)(O)(H_2_O)]^2+^ (BQCN=N,N′-dimethyl-N,N′-bis(8-quinolyl)cyclohexanediamine), [Mn^IV^(OH)_2_(H,MePytacn)] and [Mn^IV^(O)(OH)(H,MePytacn)]^+^ (Pytacn=N,N′-dimethyl-N,N′-bis(2-pyridylmethyl)-cyclohexane-trans-1,2-diamine) towards activation of C-H bonds of alkyl-functionalized aromatic molecules and the oxidation of aromatic substrates, alkenes and benzyl alcohol [117–120]. The dimerisation of the highly reactive oxo-manganese(IV) complex has been observed in the case of [(Bn-TPEN)Mn^IV^O]^2+^ (Bn-TPEN=N-benzyl-N,N′,N′-tris(2-pyridylmethyl)-1,2-diaminoethane) and the HAT reaction of anthracene and anthraquinone also studied [121–124]. Also the effect of non-redox active Sc^3+^ ion on the stability of the Mn(IV)=O species such as [(Bn-TPEN)Mn^IV^(O)]^2+^ and [(N4Py)Mn^IV^(O)]^2+^ (N4Py=N,N-bis(2-pyridylmethyl)-*N*-bis(2-pyridyl)methylamine) are studied and found that the formation of Mn^IV^(O)–(Sc^III^) complexes and observed that these scandium bound high-valent species catalyse the sulfoxidation of thioanisoles by direct oxygen atom transfer from Mn^IV^(O) complexes whereas without Sc^3+^ ion involved in electron-transfer reaction rather than OAT [124, 125]. Talsi and coworkers studied the epoxidation of olefins with various oxidants (CH_3_CO_3_H vs. *m*-CPBA, *t*-BuOOH vs. cumyl hydroperoxide, PhIO vs. iodosylmesitylene) using non heme aminopyridinylmanganese(II) complexes [LMn^II^(OTf)_2_] as catalysts and high-valent intermediate species [LMn^IV^O]^2+^ and [LMn^IV^(µ-O)_2_Mn^III^L]^3+^ were detected upon the interaction of complex with oxidants by EPR techniques [126]. Feringa et al. studied the mechanism of *cis*-dihydroxylation and epoxidation of alkenes catalysed dinuclear manganese complex [${\text{Mn}}_{2}^{\text{IV}}$(μ-O)_3_(tmtacn)_2_]^2+^ with triazacyclononane ligand framework using H_2_O_2_ as mild oxidant [127]. Interestingly, high turnover enantioselective alkene *cis*-dihydroxylation is achieved with H_2_O_2_ based on the chiral carboxylate ligands on the manganese complexes and the reactivity and selectivity is readily tunable by variation of the carboxylic acid employed. The preference of the [${\text{Mn}}_{2}^{\text{III}}$(µ-O)(µ-RCO_2_)_2_(tmtacn)_2_]^2+^ catalyst systems towards electron-rich *cis*-alkenes with high turnover numbers and efficiency demonstrated that this could be a sustainable and synthetically useful method with H_2_O_2_ as the terminal oxidant [128, 129]. Similarly the Mn(IV) complex [${\text{Mn}}_{2}^{\text{IV}}$(μ-O)_3_(Me_3_tacn)_2_]^2+^ with substituted ligands can greatly promote the alkene epoxidation efficiency under mild conditions with H_2_O_2_ and identified the active intermediate species HO-Mn^III^-(μ-O)-Mn^IV^=O or O=Mn^IV^-(μ-O)-Mn^IV^=O spectroscopically [130]. Choe et al. explored the catalytic reactivity of di-μ-oxo-bridged diamond core complexes Mn^III^-(μ-O)_2_-Mn^IV^ by adding non-redox metal ions to dissociate those dimeric cores and provided clues to understand the mechanism of methane monooxygenase which has a similar diiron diamond core as the intermediate [131]. Kwong et al. detected manganese(IV)-oxo porphyrin upon the reaction of the manganese(III) porphyrin with PhI(OAc)_2_ and excellent catalytic efficiency with up to 10,000 TON was achieved for epoxidation of olefins and proposed the involvement of manganese(V)-oxo intermediate as the premier active oxidant in the catalytic cycle [132]. Recently, Dai et al., demonstrated that manganese complex with a porphyrin-like ligand catalyzes the highly chemoselective and enantioselective oxidation of heteroaromatic sulphides with hydrogen peroxide in good to excellent yields with very high enantioselectivities (up to 90% yield and up to > 99% ee) and proposed high-valent Mn^IV^-O· radical as the reactive oxidant in the catalytic cycle [133]. Shulpin and coworkers reported the efficient oxygenation of alkanes with H_2_O_2_ catalysed by a binuclear manganese(IV) complex [Mn_2_L_2_O_3_]^2+^ (L = 1,4,7-trimethyl-1,4,7-triazacyclo-nonane) with carboxylic acid as a co-catalyst. The transformation of alkane into the corresponding alkyl hydroperoxide proceeds via generation of alkyl radicals which is rapidly react with atmospheric molecular oxygen [134]. Also the catechol oxidase activity involves HAT by the high-valent bis(oxo)-bridged manganese(IV) complex reported by Mondal group [135]. High-valent Mn(IV)-oxo intermediates mediated oxidation reactions such as epoxidation and *cis*-dihydroxylation of olefins, alkane oxidation, sulphoxidation and hydrogen abstraction are realised in many molecular systems and in some cases it is promising, however the issues such as stability and selectivity and use of strong oxidant are to be solved for the real industrial application of these molecular systems.

### High-valent Mn^V^-oxo Intermediates at Molecular Sites

The Mn^V^=O species are considered to be involved in many HAT and OAT reactions during the reaction with manganese(III) complexes with dioxygen or oxidants such as hydrogen peroxide (H_2_O_2_) or *tert*-butylhydrogenperoxide (*t*-BuOOH) or *m*-chloroperbenzoic acid (*m*-CPBA) and substrate (Table 2). Initially, Kochi group studied the catalytic activity of the various substituted Mn^III^(salen) complexes with iodosylbenzene (PhIO) towards alkenes and studied the interaction by uv–visible spectroscopy [136]. Even though they proposed the involvement of Mn^V^=O species in the catalytic activity there were no solid evidence has been collected. Collins group generated and characterised the first stable oxomanganese(V) complex by utilising a tetra-anioic ligands with two amido nitrogens and two alkoxide oxygen donors and upon reaction of the Mn(III) complex with *t*-BuOOH and found that the high-valent species are not stable in water [137]. Followed by these study the same group generated another Mn(V)=O species with tetraamido ligands which is stable in water and characterised by XRD and IR and Raman studies. However, the Mn^V^=O species is stable at room temperature and do not undergo oxygen-atom-transfer reactions possessing diamagnetic (low-spin d^2^ configuration) configuration [138]. Later on, Jacobsen group reported the manganese salen-type catalysts for the asymmetric epoxidation of unfunctionalized olefins with PhIO as oxidant with moderate enantioselectivities for a few substrates (e.g. trans-alkenes, terminal alkenes) and proposed the involvement of Mn(V)=O species [139]. In 1992 Collins group again characterised the Mn(V)=O species by using isotopic labelling and Raman spectroscopic studies [140]. ƠHalloran group generated the high-valent Mn(V)=O species along with hydroxylated solvent molecules upon reaction of the tetraanionic Mn(III) complex with molecular oxygen and they also characterised the species using UV–visible spectroscopy [141]. In the year of 1997, Groves and co workers reported the successful characterisation of Mn(V)=O species using substituted porphyrin ligands and *m*-CPBA and observed a characteristic signal in UV–visible spectroscopy [142]. Plattner et al. first time observed the direct evidence for the involvement of high-valent Mn(V)=O species towards OAT of olefins and sulphides by reaction of Mn(salen) complexes with *m*-CPBA and confirmed by electrospray tandem mass spectrometry [143]. In 1998, Collins again reported the reactivity of the Mn(V)=O complexes of tetraamido ligands in which they activated the sluggish complexes by adding alkali and alkaline earth secondary metal ions and observed OAT towards olefins [144]. However, Groves observed that addition of sodium nitrite to the low-spin, d2 oxo-Mn(V) species leads to the formation of high-spin Mn(IV) and Mn(III) states in which an electron is promoted from dxy to d(xz, yz) because of the vibronic effect due to the elongation of the very short Mn-oxo bond upon reduction [145]. Also they observed the rapid and reversible oxygen atom transfer from the Mn(V)-oxo species to bromide ion [146]. Talsi et al. extensively studied and characterised the Mn(IV/V)=O species of Mn(salen) complexes generated with various oxidants using NMR and EPR techniques [110]. Nam et al. isolated oxomanganese(v) porphyrin intermediate with H_2_O_2_, in buffered aqueous solution and demonstrated as active epoxidizing intermediate in the catalytic epoxidation of olefins. Interestingly they observed the pH dependence of the O–O bond cleavage in the mechanism with various hydroperoxides [147]. The intermediate Mn^V^=O species generated from the corresponding Mn(III) complex with H_2_O_2_ has been identified with Me_3_TACN ligands using ESI–MS and studied their role in HAT reactions of phenols as one-electron reductants by Oakes group [148]. After that Collman group studied the stereoselectivity of olefin epoxidation catalyzed by Mn^III^(salen) complexes in the presence of neutral donor ligands, employing various iodosylarenes (ArIO: PhIO, C_6_F_5_IO, and MesIO) as the oxygen atom source and single Mn^V^(salen)-oxo species is considered to be involved as the sole oxygenating intermediate [149]. Goldberg and co-workers successfully generated the high-valent Mn^V^=O species upon reaction of the Mn(III) Triazacorrole complex with PhIO and utilised first time for the sulphoxidation of thiols [150]. In 2007, Nam group generated the Mn^V^=O generated with Mn(III)-porphyrin and PhIO and characterized with various spectroscopic techniques such as UV–vis, EPR, ^1^H and ^19^F NMR, resonance Raman, and X-ray absorption spectroscopy and found that it is not active towards olefins but involved in OAT of thioanisole and triphenylphosphine [151]. The same group again generated both Mn^V^– and Mn^IV^–oxo porphyrins in basic aqueous solution and investigated their reactivities in C–H bond activation of hydrocarbons. Also they found that the C–H bond breaking ability of Mn^V^–oxo complex is 150 times faster than that of a Mn^IV^–oxo complex in the oxidation of xanthenes [152]. Chang group synthesised the Mn^V^=O species of highly bulky bis-pocket corrole 5,10,15-tris(2,4,6-triphenylphenyl)corrole (H_3_TTPPC) with PhIO and characterised the triply bonded Mn≡O moiety by Resonance Raman spectroscopy in manganese(V)–oxo complex and performed the direct oxygen atom transfer from (TTPPC)Mn≡O to styrene confirmed by an ^18^O-labeling experiment. The (TTPPC)Mn^III^ complex also exhibits significant shape selectivity in the catalytic epoxidation of nonconjugated dienes [153]. Later for the first time trans-dioxomanganese(V) porphyrin complexes have been synthesized by Nam group and employed successfully for hydride transfer from dihydronicotinamide adenine dinucleotide (NADH) analogues proceeds via proton-coupled electron transfer, followed by rapid electron transfer [154]. Goldberg isolated the high-valent metal-oxo and metal–imido complexes manganese corrolazines (TBP_8_Cz)Mn^V^(O) and (TBP_8_Cz)Mn^V^(NMes) and employed as catalysts for epoxidation of alkenes with ArIO oxidants In which the metal-imido complex is a rare example of oxygen atom transfer catalyst and these reactions likely proceed via an unusual ArIO–metal–oxo/imido intermediate [155]. Interestingly, Fukuzumi et al. generated the Mn^V^=O species by using [Ru(bpy)_3_]^3+^ as electron transfer agent and water as oxidant and catalyses alkenes and alkanes and produced epoxides and diols and aldehydes [156]. For the first time Borovik group generated the high-spin oxomanganese(V) species in a trigonal geometry unlike the other low-spin species in tetragonal geometry and characterised by EPR spectroscopy. The high-spin oxomanganese(V) complex formulation and oxidation reaction with [FeCp_2_]^+^ were monitored using optical and EPR spectroscopies [67]. Goldberg studied the effect of lewis acid Addition of anionic donors to the manganese(V)–oxo corrolazine complex MnV(O)(TBP8Cz) has a dramatic influence on oxygen-atom transfer (OAT) reactivity with thioether substrates provides new, fundamental insights regarding the influence of axial donors on high-valent Mn^V^(O) porphyrinoid complexes. Lei et al. generated the iodosylarene metalloporphyrin adducts and manganese(V)-oxo porphyrin species as a cytochrome P450 analogue and studied the mechanism of OAT with *cis*-stibene using UV-visble and ESI–MS analysis [157]. Interestingly, Goldberg activated the high-valent Mn^V^=O species of corrolazine using non-metallic lewis acid and axial coordination of the anions for the high reactivity towards thioether substrates [158, 159]. Interestingly, Neumann observed the formation of O_2_ in water catalysed by a polyfluorooxometallate with Mn(IV)-OH and Mn(V)-OH center confirmed by EXAFS measurement [160]. Recently, Nam group successfully generated the mononuclear non-heme manganese(V)–oxo complex [Mn^V^(O)(TAML)]^−^ synthesized by activating dioxygen in the presence of olefins with weak allylic C–H bonds confirmed by various spectroscopic and crystallographic analysis. Also, they studied the interaction of a Mn(V)–oxo complex with Sc^3+^ ion and found that the binding site of the Sc^3+^ ion is TAML ligand not the Mn–O moiety [161]. Hayashi group studied the myoglobin reconstituted with a manganese porphycene and found that the engineered myoglobin serves as an artificial metalloenzyme for inert C–H bond activation such as oxidation of ethyl benzene via a high-valent Mn^V^=O species similar to the species employed by native monooxygenases such as cytochrome P450A [162]. Very recently, Goldberg reported the hydrogen atom transfer reactivity of the Mn^V^=O species with phenol and also they identified the generation of Mn^IV^-OH species in the pathway of formation of the final Mn(III)-OH_2_ complex by abstraction of stepwise abstraction of two hydrogen atoms [163].

**Table 2:** List of Mn^V^ = O species generated and characterised with different ligands

Year	Structure of Mn^V^=O species	Condition	Characterisation	References
1989		TBHP, THF, *t*-BuOH and H_2_O	NMR and XRD	[137]
1990		TBHP, THF	NMR, IR and XRD, Raman	[138]
1994		O_2_, Bu_4_NCl, THF, −50 °C	UV–visible, XRD	[141]
1997		*m*-CPBA	UV–visible	[142]
1997		PhIO	ESI–MS	[143]
2002		*t*-BuOOH or H_2_O_2_ or *m*-CPBA, pH = 10.5, 0 °C	ESI–MS	[147]
2004		H_2_O_2_, H_2_O	ESI–MS	[148]
2004		*m*-CPBA, DCM	Raman, UV–visible	[150]
2005		*m*-CPBA	UV–Vis, EXAFS, XRD	[159]
2007		*t*-BuOOH or H_2_O_2_ or *m*-CPBA	UV–Vis, EPR, ^1^H and ^19^F NMR, Raman and EXAFS	[151]
2009		2 equiv. H_2_O_2_ and 2 equiv. *t*-BuOOH	UV–visible	[152]
2009		PhIO	UV–visible and Raman	[153]
2009		*m*-CPBA, Bu_4_NOH	UV–visible ESI–MS	[154]
2012		[FeCp_2_]^+^, THF/DMF −80 °C	EPR	[67]
2017		*m*-CPBA, Phosphate buffer, 10 °C	EPR, UV–visible	[162]

## Conclusion and perspectives

In this review, recent developments in natural systems operating high-valent Mn-oxygen intermediates for the catalytic reactions are focussed and how their inspiration are realised in nano and molecular levels are discussed. In OEC and ribonucleotide reductases high-valent Mn-oxo intermediate catalyse the O–O bond formation or radical generation respectively. In both the natural catalysts the active sites are stabilised by ligands from protein side chains which dictate the overall mechanism and stabilise the high-valent Mn-oxo intermediates to achieve the high activity and selectivity of the reaction. Inspired from these processes, as presented in the review several artificial systems have been synthesised and utilised for different oxidation reactions in which the oxygen atom transfer (OAT) or hydrogen atom transfer (HAT) mechanism is suggested. However, the stability and selectivity of the synthetic complexes are necessarily to be improved to overcome the stability issues and its catalytic function to the level of industrial production of energy and other organic materials. Inspired from the natural system, the heterobimetallic approach should be considered to induce high-level distortion in the active site to mimicking the function of the OEC and should be extended to various oxidation reactions. Only Mn^IV^=O species are realised in nano particles however OEC complex using either Mn^IV^-O· or Mn^V^=O species for the O–O bond formation. So the realisation of these species and use of Mn^IV^=O or Mn^V^=O species for various OAT and HAT reactions on the manganese oxide nano particles should be considered, so that the strategies learned from the natural systems to be successfully applied for the real application of the mankind.
